# Case report: Exploration of abrocitinib in the treatment of refractory chronic spontaneous urticaria: a case series

**DOI:** 10.3389/fimmu.2024.1466058

**Published:** 2024-10-14

**Authors:** Na Du, Dan Wang, Jingyi Yang, Yiwen Zhang, Xinyan Lyu, Wei Min, Sicheng Zhao

**Affiliations:** ^1^ Department of Dermatology and Venereology, The First Affiliated Hospital of Soochow University, Suzhou, China; ^2^ Department of Dermatology and Venereology, Suzhou Ninth Hospital Affiliated to Soochow University, Suzhou, China; ^3^ Department of Dermatology and Venereology, Suzhou Kowloon hospital, Shanghai Jiao Tong University School of Medicine, Suzhou, China

**Keywords:** JAK inhibitors, abrocitinib, omalizumab, chronic spontaneous urticaria, refractory urticaria, efficacy and safety

## Abstract

In clinical practice, some cases of chronic spontaneous urticaria (CSU) remain difficult to treat, with up to 40% of patients showing no response to even high (4-fold) daily doses of antihistamines. Approximately 30% of CSU patients fail to achieve complete control and relief through treatment with omalizumab and may require alternative therapies. Abrocitinib is a small-molecule oral JAK1 inhibitor that suppresses intracellular signaling of multiple key cytokines involved in inflammation cascades, and has shown beneficial effects in patients with mast cell activation disorders. We conducted a retrospective analysis of the diagnosis and treatment records of adult patients with refractory CSU who were treated with abrocitinib after inadequate response to omalizumab (defined as no response to 300 mg/4 weeks of omalizumab treatment for 6 months, followed by adjustment to 300 mg/2 weeks for a further 3 months without controlling symptoms). We also collected data on relevant treatment modalities, clinical outcomes, and adverse events. Among these patients, various treatment modalities failed to adequately control symptoms, but switching to abrocitinib significantly improved clinical outcomes. Therefore, abrocitinib may represent a new treatment option for patients with refractory CSU.

## Introduction

Urticaria is a type of mast cell-mediated disease characterized by wheals with or without angioedema. Urticaria lasting more than six weeks is called chronic urticaria (CU) (1). According to the classification of inducing factors, CU is divided into chronic spontaneous urticaria (CSU) and chronic inducible urticaria, of which CSU accounts for a higher proportion, about 60%-90% of CU (1, 2). CSU is more common in middle-aged people over 30 years old, and women are more affected than men. In 2011, the reported point prevalence of CSU in Europe was 0.5%-1.0% (3). In 2019, the proportion of CSU patients among new college students was 2.7% in China (4). CSU has a significant impact on patients’ quality of life, such as sleep (5), and imposes a heavy economic burden on both patients and society due to long-term treatment (6–8). Standard doses, increased doses, or combination second-generation antihistamines are the treatment of choice for CSU. However, after one year of treatment with second-generation antihistamines, up to 42.2% of patients still suffer from uncontrolled symptoms (9). In recent years, although omalizumab has been increasingly used to treat refractory CSU, about 30% of CSU patients still cannot achieve complete control and relief through omalizumab treatment. For these patients, early upgrading of treatment is recommended (10).

JAK kinase inhibitors, as a type of small molecule targeted drug, mainly affect many cytokines’ functions by inhibiting the JAK-STAT pathway, thereby regulating biological effects such as immune responses, cell differentiation, proliferation, and apoptosis (11). In current medical practice, JAK inhibitors are gradually becoming more widely used. Despite this, data on the clinical efficacy and safety of JAK inhibitors in refractory CSU after omalizumab treatment failure are limited. This case series aims to provide real-world clinical results of abrocitinib treatment for patients with refractory chronic urticaria who have failed omalizumab treatment to assist clinical doctors.

## Case presentation

Patient 1, a 38-year-old female who has been suffering from chronic spontaneous urticaria (CSU) and angioedema for a year.The patient consistently presents with erythema and urticaria on the trunk and extremities, accompanied by angioedema of the lips and eyelids. 7-day urticaria activity score (UAS7) was 34.Despite being treated with the maximum dose of second-generation antihistamines for four weeks, her condition did not improve. After consultation, she was given omalizumab at a dose of 300mg every four weeks for six months, but her symptoms did not improve. The dosage was then adjusted to 300mg every two weeks and she continued treatment for an additional 12 weeks, but her urticaria and angioedema did not improve.

Since previous treatments were ineffective, omalizumab was discontinued and she was given abrocitinib (Xeljanz, Pfizer) 100mg orally once a day. In the two weeks of abrocitinib treatment, the patient had four urticaria attacks and one episode of angioedema on her upper lip and eyelids. UAS7 was 15. Due to the patient’s long-term suffering from CSU and the eagerness to control the condition promptly, after thorough communication with the patient, she was treated with systemic corticosteroids for three days. With continued abrocitinib treatment, the patient’s CSU symptoms disappeared completely, and she did not experience any angioedema attacks during the subsequent treatment period. UAS7 was 3.After a month, the abrocitinib dose was reduced to once every other day, and the patient discontinued treatment after three months when her condition had completely improved. UAS7 was 0.There were no adverse reactions observed, and during the one-year follow-up period, there were no relapses.

Patient 2, a 41-year-old male, was diagnosed with chronic spontaneous urticaria (CSU) 1.5 years ago.The patient presents with erythema and urticaria on the face, neck, trunk and extremities 5 days a week.He had a history of alopecia areata and allergic rhinitis. The patient had previously undergone various treatments, including oral antihistamines (cetirizine 10mg and levocetirizine 10mg, up to four times the normal dose), omalizumab, prednisolone, cyclosporine and methotrexate. Due to incomplete control of symptoms and adverse reactions to some drugs, the patient agreed to start treatment with 100mg/day of abrocitinib after being informed of the risks.

Before starting abrocitinib the patient’s UAS7 was 40. After two weeks of abrocitinib treatment, urticaria symptoms, including wheals and itching, gradually improved, and UAS7 decreased to 15. After three months of continuous treatment, wheals and itching disappeared completely. UAS7 was 0. The dosage was then gradually reduced to 100mg every other day for one month, with no adverse reactions. Furthermore, the patient reported relief from symptoms of alopecia areata and allergic rhinitis. There was no recurrence of the patient’s condition after being lost to follow-up.

Patient 3, a 26 year old female, had chronic spontaneous urticaria(CSU) for 2 years. The skin appears as a diffuse swelling on the head, neck, trunk, and limbs.UAS7 was 30.She had a previous history of allergic rhinitis. Patient with the maximum doses of H1 and H2 antihistamines, montelukast, and omalizumab 300 mg/2w were incompletely controlled. After withdrawal of omalizumab, oral abrocitinib 100 mg/d was started with significant reduction of symptoms within 2 weeks,and UAS7 decreased to 6. Abrocitinib was used for 3 months and had no adverse effects during the medication. After complete resolution of symptoms (UAS7 was 0) and 6 months of withdrawal, the disease recurred and H1 and H2 antihistamines were restarted for 2 weeks. In the absence of improvement, oral abrocitinib 100 mg/d was started and remission was reached again. The patient developed mild transaminase elevation 6 weeks after resuming abrocitinib and improved after symptomatic treatment. Abrocitinib was used for 8 months. No recurrence after follow-up after 1 year.

Patient 4, a 45-year-old female, had a history of hypertension, asthma, and hyperthyroidism with a 2-year CSU. The patient presented with diffuse swelling and pronounced itching. UAS7 was 36. The disease did not respond to maximum doses of H1- and H2-antihistamines, Montelukast, hydroxychloroquine, and omalizumab 300 mg/4w for 6 months. When omalizumab was discontinued and cyclosporine 1 mg/kg/d was started for 1 month, symptoms were partially controlled, the dose of cyclosporine was increased to 3 mg/kg/d, urticaria was almost completely improved, and gradually reduced to 0.5mg/kg/d with remission. After 3 months, the patient developed poor blood pressure control with previous antihypertensive drugs, hyperglycemia, hematuria, and hyperuricemia, so cyclosporine was discontinued, and after the patient’s blood pressure, blood glucose, blood uric acid, and hematuria returned to normal, oral abrocitinib treatment was changed to 100mg/d. One month later, the patient’s symptoms were still in continuous remission, UAS7 was 7 and no significant abnormalities were observed in blood pressure, blood sugar, uric acid, etc., which is still under follow-up.

Patient 5, a 36-year-old male with a 1-year history of CSU and experienced with diffuse swelling four or five days a week. UAS7 was 25. It was unable to control his disease with the highest previous doses of H1 and H2 antihistamines. Three months ago, omalizumab was given 300mg/4w three times due to obvious aggravation of wind masses and pruritus. The patient complained that after each omalizumab injection, the condition worsened significantly and was accompanied by chest discomfort. In the past one month, the patient took prednisone 25mg qd and tripterygium glycoside 20mg tid to control the disease. Because the patient had a birth plan for nearly half a year, after informed consent, the treatment was adjusted to oral abrocitinib 100mg/d, which has been used for 1 month and symptoms have improved to a great extent without adverse reactions. UAS7 decreased to 5. Patients who have been asked to return after 2 weeks can be reduced and maintained for 1 month during follow-up.

Patient 6, a 56-year-old female, suffered from chronic spontaneous urticaria (CSU) for five years and experienced wind clusters almost every day. UAS7 was 28. To control the condition, the patient took high doses of oral antihistamines and intermittently administered oral or intramuscular corticosteroid hormones. The patient also received immunosuppressive treatments such as methotrexate and cyclosporine. However, due to poor response to previous medications and the possible adverse effects of long-term use of immunosuppressants, abrocitinib (100mg once daily) was given after discussing with the patient and their family. During the second month, the patient’s urticaria symptoms gradually improved, UAS7 was 7 and the previous medication was gradually decreased. The abrocitinib treatment lasted for three months and was discontinued when the symptoms disappeared. The clinical characteristics of the six patients aresummarized in **Table 1** as follows.

**Table 1:** Clinical characteristics of the patients.

Patient	Sex/age	Comorbidities	Disease duration	Skin biopsy	Diagnosis	Previous therapies	Abrocitinib dose	Duration of treatment with abrocitinib	Current status	Side effects
1	F/38	None	I year	N/P	CSU, angioedema	AHs,Omalizumab, Cs	100mg ,qd	For 3 months	Complete remission/no relapse after I year	None
2	M/41	Alopecia areata and allergic rhinitis	1.5 year	N/P	CSU	AHs,Omalizumab, Cs,CsA,MTX	100mg,qd	For 4 months	Complete remission	None
3	F/26	Allergic rhinitis	2 year	N/P	CSU	AHs,Omalizumab, Mendrust	100mg ,qd	For 8 months	Complete remission/no relapse after 1 year	None
4	F/45	Hypertension, asthma,and hyperthyroidism	2 year	N/P	CSU	AHs,Omalizumab, Mendrust,CsA,Hyd roxych-loroquine	100mg,qd	Since 2 months ago till now	Significant but not complete Improvement	None
5	M/36	None	I year	N/P	CSU	AHs,Omalizumab, Cs,Tripterygium glycosides	100mg ,qd	Since 3 months ago till now	Complete remission	Nonr
6	F/56	Hypertension	5 year	N/P	CSU	AHs,Cs CsA,MTX	100mg ,qd	For 3 months	Complete remission/no relapse	None

AHs, antihistamines; QD, once a day; Cs, corticosteroids; CsA, cyclosporine; F, female; M, male; MTX, methotrexate; N/P, not performed.

**Figure 1:**
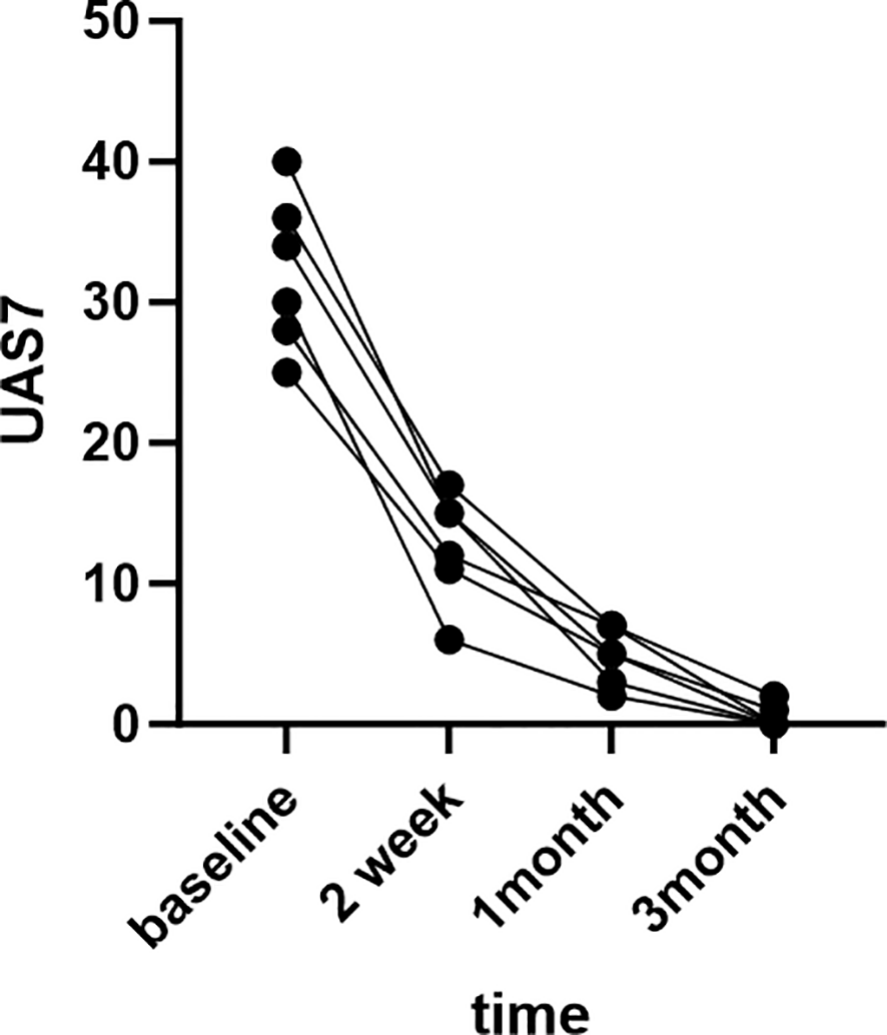
Therapeutic outcomes of six patients.

The patients were very satisfied with the treatment results and expressed their gratitude to us.The treatment outcomes of six patients are depicted in the **Figure 1** below.

## Discussion

Janus kinases (JAKs) are a class of intracellular non-receptor tyrosine kinases composed of JAK1, JAK2, JAK3, and TYK2 subtypes. It is associated with signaling downstream of various type I and type II cytokines (such as IL-6, IL-17, IL-19, IL-20, IL-22, IL-23, IFN-γ, etc.) receptors. These cytokines and signaling pathways are involved in biological processes such as immune cell differentiation, maturation, humoral immune regulation, immune barrier function, cell proliferation, and apoptosis, and play a key role in autoimmune, allergic, and inflammatory disease responses (11). Abrocitinib is a selective inhibitor of JAK1 that reversibly and selectively inhibits JAK1 by blocking the adenosine triphosphate binding site. On April 8, 2022, Abrocitinib was approved by the China National Medical Products Administration. For the treatment of adults with moderate-to-severe atopic dermatitis who do not respond well to other systemic therapies (such as hormones or biologics) or who are not appropriate for existing therapies. However, our findings suggest that abrocitinib may be a new option for treating refractory CSU in adults who have not responded well to omalizumab.

According to the expert consensus on the treatment of chronic spontaneous urticaria in China (2023 edition), if the CSU symptoms cannot be completely controlled by increasing the dose of omalizumab or shortening the interval of treatment for 12 weeks, it is recommended to replace or add cyclosporine, methotrexate, azathioprine, sulfoxone, glucocorticoid, Janus kinase inhibitor, tripterygium preparation and other traditional Chinese medicines for treatment. Some patients stopped taking traditional immunosuppressants because of delayed response, poor efficacy, or serious adverse reactions, and there were easy recurrences after withdrawal regardless of the treatment method used.

At present, the mechanism of action of JAK inhibitors in urticaria remains to be clarified. The pathological mechanism of CSU suggests that mast cells and basophils are the main effector cells. At present, it is known that two types of autoimmune reactions, type I and type IIb, are mainly involved in mast cell activation in CSU patients, resulting in mast cell degranulation and release of inflammatory mediators, increase skin vascular permeability, activate nerve fibers in skin tissue, and cause symptoms such as wind mass and pruritus (12). Mast cells are activated by anti-Fcepsilonri or anti-IgE autoantibodies; however, the poor response observed in some patients to anti-IgE drugs such as omalizumab indicates that mast cell activation mechanisms go beyond high-affinity IgE receptor stimulation (13). The new pathogenesis suggests that CSU is an immune disorder caused by changes in the cytokine-chemokine network. Recent studies have found that up-regulation of IL-9 and IL-10 is conducive to the development of CSU. Feng et al. ‘s results showed that IL-9/IL-10 is involved in the pathogenesis of CSU through the JAK/STAT signaling pathway, and inhibition of the JAK/STAT signaling pathway can effectively inhibit the activity of the disease, thus enabling effective treatment of CSU (14). This is consistent with our case series.

In terms of safety, there have been few reports of serious adverse events in patients treated with abrocitinib for skin diseases. In our study, abrocitinib was well tolerated. One patient experienced mild dizziness after the first dose, and one patient experienced mild aminotransferase elevation after the first dose. Symptomatic treatment was given, but the above symptoms gradually disappeared with the extension of medication time. No adverse reactions were observed in the remaining patients. All patients were screened for latent TB hepatitis or HIV before starting abrocitinib.

In conclusion, abrocitinib is effective in patients with refractory CSU and could be a new, safe treatment option for adults with refractory CSU. I believe that for patients with refractory CSU who do not respond well to traditional treatment and have an inadequate reaction to omalizumab, abrocitinib can be considered as an alternative treatment for refractory CSU after ensuring that there are no contraindications associated with its use. We have to point out that our patients’ number was limited and bias may exist. Regrettably, it was not feasible to capture clinical photographs of each patient before and after treatment with abrocitinib due to the irregular location and timing of urticaria lesions.Further research and clinical studies are warranted to corroborate these findings and establish the long-term safety and efficacy of abrocitinib in managing CSU.

## Data Availability

The original contributions presented in the study are included in the article/supplementary material. Further inquiries can be directed to the corresponding authors.
